# A Role for the Biological Clock in Liver Cancer

**DOI:** 10.3390/cancers11111778

**Published:** 2019-11-11

**Authors:** Gianluigi Mazzoccoli, Luca Miele, Giuseppe Marrone, Tommaso Mazza, Manlio Vinciguerra, Antonio Grieco

**Affiliations:** 1 Department of Medical Sciences and Chronobiology Laboratory, Fondazione IRCCS Casa Sollievo della Sofferenza, 71013 San Giovanni Rotondo (FG), Italy; 2 Fondazione Policlinico Universitario A Gemelli–IRCCS– Catholic University of the Sacred Heart, 00168 Rome, Italy; luca.miele@policlinicogemelli.it (L.M.); giuseppe.marrone@policlinicogemelli.it (G.M.); antonio.grieco@unicatt.it (A.G.); 3 Bioinformatics Unit, Fondazione IRCCS Casa Sollievo della Sofferenza, 71013 San Giovanni Rotondo (FG), Italy; t.mazza@css-mendel.it; 4 International Clinical Research Center (FNUSA-ICRC), St. Anne’s University Hospital, 65691 Brno, Czech Republic

**Keywords:** hepatocellular carcinoma (HCC), circadian clock, chronotherapy

## Abstract

The biological clock controls at the molecular level several aspects of mammalian physiology, by regulating daily oscillations of crucial biological processes such as nutrient metabolism in the liver. Disruption of the circadian clock circuitry has recently been identified as an independent risk factor for cancer and classified as a potential group 2A carcinogen to humans. Hepatocellular carcinoma (HCC) is the prevailing histological type of primary liver cancer, one of the most important causes of cancer-related death worldwide. HCC onset and progression is related to B and C viral hepatitis, alcoholic and especially non-alcoholic fatty liver disease (NAFLD)-related milieu of fibrosis, cirrhosis, and chronic inflammation. In this review, we recapitulate the state-of-the-art knowledge on the interplay between the biological clock and the oncogenic pathways and mechanisms involved in hepatocarcinogenesis. Finally, we propose how a deeper understanding of circadian clock circuitry–cancer pathways’ crosstalk is promising for developing new strategies for HCC prevention and management.

## 1. Introduction

Worldwide, liver cancer ranks second among the principal causes of cancer-related death and fifth in men and ninth in women among the most commonly diagnosed cancers, respectively, with more than 800,000 new cases in 2018 and hepatocellular cancer (HCC) accounting for 70–85% of all liver cancers [1,2]. Recent availability of effective direct antiviral agents targeting hepatitis C virus (HCV) NS3/4A (protease), NS5B (polymerase), and NS5A (nonstructural) protein has greatly curtailed the causative role of HCV infection in hepatocarcinogenesis and the most important risk factor for liver cancer is actually represented by excess body fat. The global epidemics of obesity, metabolic syndrome, type 2 diabetes, and nonalcoholic fatty liver disease (NAFLD) facilitated the quick rise in HCC prevalence [3]. NAFLD may progress to nonalcoholic steato-hepatitis (NASH), hallmarked by steatosis with necroinflammation, and in due course to fibrosis, cirrhosis, and HCC. For HCC developing without cirrhosis, associated factors may include inflammatory, metabolic, endocrine, bile acid flux, and gut microbiome derangements associated with obesity and liver fat accumulation [3].

## 2. The Circadian Clock Circuitry and the Molecular Mechanisms of Hepatocellular Carcinogenesis

Hepatic carcinogenesis is linked to the ongoing failure of mechanisms managing basic cellular processes, such as cell cycle, proliferation, differentiation, apoptosis, DNA damage response, autophagy, xenobiotic detoxification, anabolic/catabolic processes, and oxidation-reduction reactions with reactive oxygen species production/antioxidant defenses balance [4]. Key embryogenesis-related and oncogenic pathways have been recognized by genetic studies as deranged as well, among which WNT/β-catenin, proliferation, and hepatoblastoma-like pathways, at present are not easily druggable for targeted cancer therapy [4]. The greater part of the aforementioned biological processes and signaling pathways are hallmarked by rhythmic activity fluctuations with about 24-h (circadian) periodicity [5,6,7].

The nycthemeral rhythmicity featuring behavior (locomotor activity, eating/fasting, sleeping/waking) and physiology (temperature, blood pressure, hormone secretion) of living beings is controlled by the circadian timing system. This time-keeping system is organized as a hierarchical network comprising self-sufficient biological oscillators in the hypothalamic suprachiasmatic nuclei (SCN) and in peripheral tissues. The circadian timing system operates by transduction and integration of photic signaling (outdoor light levels/indoor lighting conditions) and grants organism/species survival advantage through appropriate anticipation of expected environmental changes. The central pacemaker (SCN) entrains peripheral tissues oscillators by means of cues, such as hormone (cortisol, melatonin), physical (temperature fluctuations), and neural (autonomic nervous system fibers) outputs. This complex and rhythmic signaling drives behavioral cycles (food craving and intake, rest-activity), nervous, cardio-vascular, gastro-intestinal, and musculoskeletal system function in synchrony with metabolic pathway activation, transcriptome-metabolome oscillations, oxidation-reduction reactions, and nutrient level fluctuations [5,6,7].

At the cellular level 24-h rhythms are generated by a molecular mechanism maneuvering transcription-translation feedback loops hard-wired by intertwining circadian genes and proteins, precisely ARNTL/2 (BMAL1/2), CLOCK (or its paralog NPAS2), PERIOD (PER) 1–3, CRYPTOCHROME (CRY) 1–2, REV-ERBs/RORs nuclear receptors and TIMELESS. The transcription factors CLOCK and BMAL1 heterodimerize, bind to E-box enhancer elements in the promoters of the genes *PER1-3* and *CRY1-2* and turn on their transcription, whereas PER and CRY protein complexes impede their transcriptional activity [8]. TIMELESS intermingles with TIPIN and deals with DNA replication and damage response, above all S-phase arrest and signaling pathways mediated by ATR-Chk1 and ATM-Chk2 [9]. The nuclear receptors REV-ERB *α*/β (encoded by *NR1D1*/*NR1D2*) and ROR α/γ control *ARNTL* rhythmic transcription competing at ROR-responsive elements (RORE) of its promoter [10,11,12]. Post-translational modifications of circadian proteins, represented by phosphorylation, SUMOylation, acetylation and deacetylation, O-GlcNAcylation [13], hold up proper functioning of the molecular clockwork. In particular, phosphorylation is operated by several protein kinases, such as casein kinase (CK)1-ε (encoded by *CSNK1Ε*), adenosine monophosphate (AMP) activated kinase (AMPK), and glycogen synthase kinase (GSK)-3β [13,14]. In addition, ARNTL is SUMOylated with circadian rhythmicity in the mouse liver [15,16]. Acetylation is managed by histone/protein acetyl-transferases, such as CLOCK, while deacetylation is activated by histone/protein deacetylases, such as the NAD+-dependent SIRT1 [17,18,19,20]. The molecular clockwork drives the rhythmic transcription of clock controlled genes, such as the PAR bZIP transcription factors DBP, TEF, HLF, which in turn drive the expression of thousands of genes, among which are cell cycle progression regulators (Cyclin D1, Cyclin A, Mdm-2, c-Myc, WEE-1, GADD45Α) and tumor suppressor genes/oncogenes as well [21,22,23,24,25]. In pre-neoplastic liver lesions of Fischer 344 rats with induced carcinogenesis through the resistant hepatocyte model and in c-Myc/TGF-α transgenic mice, up-regulation of c-Myc, cyclin D1, cyclin A, and E2F1, involved in cyclin D1-CDK4, E2F1-DP1 complexes and pRb hyper-phosphorilation was identified [26,27]. Changes of G1 to S cell cycle phase transition ensuing from these derangements take part in human HCC, as well [28]. A proper functioning of the biological clock demonstrates tumor suppressing potential, whereas circadian rhythmicity disturbance, like that provoked by shift work in humans and chronic jet lag (CJL) in animal models, is an independent risk factor for HCC: for instance, it worked as tumor promoter in mice exposed to the hepatic carcinogen diethylnitrosamine (DEN) in combination with 8-h advance of light onset every 2 days [29]. Accordingly, chronic circadian disruption and ablation of Steroid Receptor Coactivator-2 (SRC-2), a crucial metabolic transcriptional co-regulator in SCN and liver, altered behavioral activities and metabolic homeostasis in SRC-2(-/-) mice, leading to NAFLD, NASH, and HCC [30]. Furthermore, animal experiments performed in wild type and core clock genes mutated mice revealed an evident augment of early NAFLD onset with progression to NASH, fibrosis, and, in due course, HCC. The exploration of the molecular mechanisms revealed deregulation of liver metabolic genes enriching nuclear receptor-controlled cholesterol/bile acid and xenobiotic metabolism pathways and suggested a protective role for farnesoid X receptor (FXR) and a pro-tumorigenic role for constitutive androstane receptor (CAR) [31]. Interestingly, evaluation of circadian genes and protein expression in human HCC and matched non-tumor specimens showed reduced expression levels of PER1, PER2, PER3, CRY2 in HCC, with over-expression of the histone methyltransferase Enhancer of zeste homolog 2 (EZH2) and promoter methylation, but no genetic mutations [32,33], and experiments performed in vitro challenging the PLC/PRF/5 human HCC cell line with CoCl2 for 24 h at increasing concentration (50, 100, and 200 µΜ) to mimic an hypoxic environment showed an additional effect of hypoxia in circadian genes’ deregulation in expression of in HCC [34].

In Americas, Australasia, and Europe the main hepatic pathology is represented by NAFLD/NASH with possible evolution to HCC. NAFLD originates from fat buildup in the liver owing to unbalance in biological processes, all of which are hallmarked by 24-h rhythmic fluctuations driven by the biological clock: lipid metabolism, metabolites/bile acids fluxes, signaling pathways downstream of nutrients, ligand bound nuclear receptors, gastro-intestinal hormones, as well as host gut barrier-microbiota interface, autophagic and immune/inflammatory processes. Worldwide estimated prevalence of NAFLD suggests that behavior-metabolism misalignment, such as for shift-work or social jet lag, is becoming a leading risk factor for metabolic diseases. This evidence implies that proper time-qualified interconnections among metabolic processes and resting-fasting/waking-feeding cycles are always necessary to avoid NAFLD/NASH-HCC progression through lipotoxicity and metaflammation. Besides, a growing body of evidence suggests that changes in life habits in conjunction with time-restricted eating and intermittent fasting schedules could positively impact the circadian timing system and the biological clock-driven processes providing a valuable additional therapeutic strategy [35,36].

## 3. The Biological Clock and the Oncogenic Pathways Involved in Hepatocarcinogenesis

Onset and progression of cancer in the liver rely on multifaceted molecular mechanisms entailing genetic and epigenetic alterations to oncogenes and tumor suppressor genes as well as disturbed control, anomalous function, and inappropriate interaction of crucial molecular cascades, such as Wnt/β-catenin, Hedgehog, MAPK, and PI3K/AKT/mTOR signaling pathways, with the circadian clock circuitry, overall favoring metaflammation, derangement of growth and differentiation control, and in due course carcinogenesis [35,36].

### 3.1. WNT/β-Catenin Signaling Pathway

The growth factors encoded by the WNT gene family manage cell fate, proliferation, migration, polarity, and death throughout development and tissue renewal/regeneration as well as in pathophysiological mechanisms of disease, including cancer and HCC in particular [37]. Several components of this pathway are hallmarked by circadian regulation and show time-related changes with 24-h periodicity (Wnt3, Wnt10a, Lef1, β-catenin, Dvl2, TCF3/4, Fzd2/3/5, Dkk3, Sox9, Lhx2, Lgr5, Lef1, Dab2, AXIN-2) [38,39,40,41]. Rhythmic expression profiles were shown for approximately 50 genes in WNT signaling in about 40 microarray datasets from two dozen different mammalian tissues [42], and analysis of data from chromatin immune-precipitation followed by sequencing revealed that for many of them ARNTL directly drives circadian expression binding to their encoding genes’ promoters [43].

### 3.2. Hedgehog Signaling Pathway

Hedgehog (Hh) signaling is involved in the regulation of stem cell dynamics, cellular proliferation-differentiation and tissue polarity in embryogenesis, normal development, and carcinogenesis [44,45]. The Hh signaling pathway is operated by several components, including the receptor and signal transducer Smoothened (SMO), which upon activation controls Gli proteins function and elicits intracellular signaling prompting the activation of transcription factors and chunking inhibitors’ production. Lacking the Indian, Desert, and Sonic ligands, SMO is functionally stalled by Patched, encoded by *PTCH1*. Furthermore, SMO activation upholds c-Myc expression and plays a part in hepatocarcinogenesis [46,47]. A recent study performed in liver and hepatocytes from wild type and Smo^flox/flox^ and Alfp-Cre lines (SAC-KO) crossbreeds with PER2::LUC reporter mice evaluated time-qualified expression of Hh signaling and molecular clockwork cogs and evidenced a crucial interplay between HH and circadian pathways [46,47]. The expression in liver and hepatocytes of numerous Hh pathway genes (*Ihh*, *Ptch1*, *Smo*, *Hhip1*, *Fu*, *SuFu*) plus serum levels of Indian Hh oscillated with 24-h periodicity, whereas Gli proteins encoding genes fluctuated scarcely Bmal1 knock-out in hepatocytes disrupted expression of Hh pathway genes, with *Gli1* and *Ihh* up-regulation and *Ptch1* down-regulation. On the other hand, SAC-KO mice showed altered clock genes’ expression, confirmed in vitro upon modulation with *Gli1* and *Gli3* siRNAs. In addition, in hepatocytes derived from SMO-KO mice lipid metabolism related genes were severely deranged and the Hh signaling-biological clock interplay was modulated by high fat diet or fasting in a time-of-day dependent manner, hinting GLI factors mediated cooperation in upholding hepatic lipid metabolism [46,47].

### 3.3. MAP Kinase Signaling Pathways

The mitogen-activated protein kinase (MAPK) pathways are triggered by means of binding of extracellular growth factors to receptor tyrosine kinases and work through the Ras-Raf-MEK-ERK signaling cascade. Specifically, MAPK pathways are transducers of extracellular signals and stressors into cellular responses and in mammalian cells comprise three foremost families: extracellular-signal-regulated kinases (ERKs), Jun amino-terminal kinases (JNKs), and stress-activated protein kinases (p38/SAPKs). These signaling cascades are operated by three sequentially activated protein kinases and set off biological responses modulating crucial cell and tissue processes, for example proliferation, differentiation, apoptosis, growth, development, inflammation, and transformation in several oncogene and growth factor-mediated processes [48]. These factors play a key role in controlling transcription of genes encoding proteins managing vital cellular functions, for instance growth, proliferation, differentiation, and subsequently cell survival. Deranged MAPK signaling activation is drawn in a broad variety of cancers and takes place through numerous mechanisms, comprising abnormal expression and/or genetic mutations of extracellular receptor tyrosine kinases. These alterations provoke unbalanced (unrelenting/excessive) receptor activity and signal transduction in the lack of proper molecular stimulus and may set in motion amplified or unrestrained cell proliferation and cancer-associated faults in apoptosis. MAPK signaling cascades and circadian pathways interplay at various levels and cooperate to manage key biological functions involved in tumor suppression as well [49]. The interaction among homologs of MAPK and circadian clock genes was highlighted in a huge range of species, ranging from the ascomycete fungus *Neurospora crassa* to insects (*Drosophila melanogaster*), amphibians (*Rana catesbeiana*), birds (*Gallus gallus*), and mammals (*Mus musculus*, *Syrian hamster*, *Homo sapiens*) and the transcriptional control of MAPK pathway modules operated by the molecular clockwork brings about prominent changes on the signaling events and biological effects, such as the expression of downstream tissue-specific genes at the appropriate time of day [50,51,52].

### 3.4. PI3K/AKT/mTOR Signaling Pathway

The PI3K/AKT signaling pathway manages crucial cellular processes including survival, apoptosis, growth, proliferation, metabolism, and motility. Beyond its physiological role, anomalous activation of the PI3K/AKT signaling pathway is critical in numerous neoplastic diseases and supports cancer cells’ survival and proliferation, tumor propagation and resistance to treatment with chemotherapy and radiotherapy as well as early intrinsic (also known as innate) resistance or rapid cancer adjustments and late acquired resistance to targeted therapy. The serine/threonine kinase AKT includes several domains: (i) an N-terminal pleckstrin homology (PH) domain, composed of 100 amino acids that are able to act together with phosphoinositides produced by the lipid kinase phosphoinositide 3-kinase (PI3K), phosphatidylinositol (3-4-5) trisphosphate (PIP3), and phosphatidylinositol (3-4) bisphosphate (PIP2); (ii) a kinase domain, confined in the middle of the molecule and comparable to other Ser/Thr protein kinases of the AGC kinase group; (iii) a C-terminal regulatory domain of about 40 amino acids and including the hydrophobic motif, whose phosphorylation starts enzymatic activity. Once activated, AKT restrains apoptosis and growth inhibition established by TGF-β and C/EBPα, respectively, and may switch on β-catenin downstream signaling. The PI3K/AKT pathway cooperates with mammalian target of rapamycin (mTOR), a serine-threonine kinase that crucially manages mRNA translation and protein synthesis, and upholds cyclin D transcription [35,53]. Interestingly, the mTOR signaling pathway and the circadian clock circuitry interplay in several ways, in particular at the level of the molecular clockwork as well as in photic entrainment and synchronization of biological clock networks [54]. The circadian protein Per2 works as a gibbet binding tuberous sclerosis complex 1 (Tsc1), Raptor, and mTOR jointly and expressly restraining mTORC1 complex activity, while the glucagon-Creb/Crtc2 signaling pathway brings on Per2 expression leading to hepatic mTORC1 suppression in fasted mice. Accordingly, lack of Per2 protein considerably increases protein synthesis and cell proliferation and decreases autophagy [55].

### 3.5. Epigenetics

**Figure 1:**
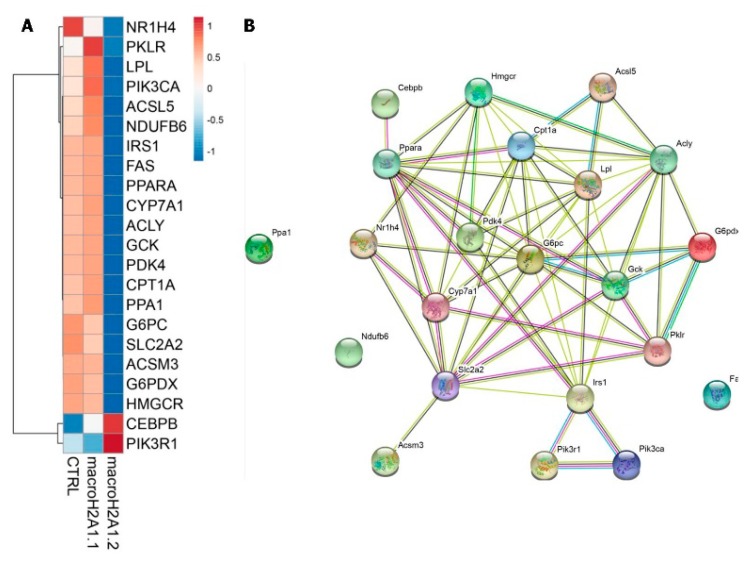
Isoform-specific impact of histone variant macroH2A1 on the expression level of circadian lipogenic genes involved in non-alcoholic fatty liver disease pathogenesis. (**A**) Heat-map rendering mRNA expression levels of 24-h oscillating genes upon free fatty acid challenge in Hepa 1-6 cells stably over-expressing histone variant macroH2A1.1 and macroH2A1.2 isoforms; (**B**) STRING interaction network wiring the 24-h oscillating genes rendered in the heat-map. Edges are colored according to the nature of the interactions between genes. Cyan and magenta links correspond to known interactions from curated databases and experimentally determined findings; green, red, and blue links correspond to predicted interactions by gene neighborhood, gene fusion, and gene co-occurrence, respectively; light green, black, and light blue links represent text mining, co-expression, and protein homology evidence of interaction, respectively. Original data from [22,75].

Epigenetics studies the hereditary modifications of gene expression not involving alterations of the DNA sequence and determining a modification in the phenotype without a change in the genotype. Usually, the epigenetic changes represent a physiological and common phenomenon, but they can also be conditioned by disparate factors including age/aging, environment, lifestyle, unhealthy habits (cigarette smoking, alcohol abuse), nutrition (even in the fetus in relation to the diet of pregnant women), or can also be determined by pathological conditions. Epigenetic modifications can represent a physiological mechanism linked to cell differentiation during embryonic development or in tissue repair/regeneration processes, or on the contrary can be part of the modifications underlying neoplastic transformation. Epigenetic modifications are progressively recognized as crucially implicated in a number of diseases, including cancer, and at the moment, at least three mechanisms of epigenetic modification have been identified, involving the initiation and maintenance of epigenetic change, in particular DNA methylation, histone modification, and gene silencing associated with non-coding RNA (ncRNA). During liver cancer insurgence and development, epigenetic modifications such as deranged methylation of DNA, are frequent phenomena [56]. As a result, the levels of DNA methylation can be used as biomarkers of the detection and prognosis of gastrointestinal cancers, including HCC, and even to predict the responsiveness to treatment [57]. Post-translational histone modifications (PTM), inclusion of histone variants, loss of genome imprinting, the remodeling of chromatin remodeling, microRNA and non-coding RNAs are additional mechanisms involved in human carcinogenesis [58]. PTM such as Histone deacetylation in the liver shows 24-h rhythmicity driven by Histone Deacetylase 3 (HDAC3), which represses circadian genes and genes managing synthesis and degradation of lipids in cells through diverse enhancer complexes. Recruitment through the NCoR–HDAC3 complex with REV-ERBα and hepatocyte nuclear factor 6 (HNF6) to lipogenic genes enhancers impacts on lipid metabolism so that Hdac3 and REV- ERBα-deficient mice display hepatic steatosis. Separately from REV-ERBα, the other liver-specific transcription factor HNF4α allows recruitment to chromatin of a different HDAC3 co-repressor complex enclosing prospero homeobox protein 1 (PROX1) [59]. HNF4α is also an effective circadian tumor suppressor, in particular its main isoform expressed in the adult liver (P1-HNF4α), whereas a different promoter driven isoform (P2-HNF4α) suppresses ARNTL and is expressed in HNF4α-positive HCC [60]. Histone variants—and in particular those belonging to the families of canonical histones H2A and H3—are recognized to confer particular features to the nuclear chromatin. Histone variants have crucial functions in modulating the lineage commitment of stem cells and the reprogramming of somatic cells to a pluripotent potential [61,62]. Changes in histone variant deposition onto chromatin affect tumorigenesis by modulating chromatin plasticity, genomic instability and cellular senescence, and triggering cancer-promoting gene expression pathways [61,62]. We recently studied the interplay between large histone variants of the macroH2A1 family and the epigenetic alterations that characterize HCC onset [63]. HCC human samples expressed higher levels of macroH2A1 proteins compared to flanking healthy liver tissue [63]. Furthermore, we observed hypomethylation of DNA in all the conditions of the hepatic disease range (NAFLD/NASH/HCC), and a tight correlation between epigenetic modifications observed in HCC and the expression of macroH2A1 isoforms [63]. Alterations in the patterns of DNA methylation are also key players leading to HCC development [64]. When HCC cells are administered with decitabine, a drug inducing DNA hypomethylation, they develop senescence and tumor proliferation is halted [63]. HCC cells over-expressing macroH2A1 proteins displayed refractoriness to decitabine-induced senescence via a pathway depending on p38 mitogen-activated protein kinase/interleukin (MAPK/IL8) [63]. However, HCC cells respond to guadecitabine—a new demethylating agent displaying a modified structure of decitabine. In guadecitabine, decitabine is stabilized by the covalent binding of guanosine [65]. In this scenario, macroH2A1 over-expression in HCC cells led to cytidine deaminase (CD) up-regulation, which in turn degraded decitabine but not guadecitabine. This reaction is related to the different chemical structures making guadecitabine five times more refractory to the degradation dependent on CD enzymes [65]. Since macroH2A1 is mechanistically involved in the differentiation of stem cells [66,67], we recently studied its implications as an epigenetic player orchestrating liver cancer stem cell (CSC) insurgence and stemness. Mice inoculated with HCC cells knock-down (KD) for macroH2A1, but not control HCC cells, displayed bigger xenograft tumors, accompanied by a low level of differentiation [68]. MacroH2A1 KD cells harbored classic features of CSC: they became chemoresistant (to doxorubicin and sorafenib, two of the most used chemotherapeutics against HCC,) and resistant to hypoxia through the production of enhanced levels of hypoxia inducible factor (HIFα1) [68]. Dissection of the cellular metabolism of macroH2A1 KD cells uncovered a massive shift toward the pentose phosphate pathway (PPP). In particular, KD cells were able to produce increased amounts of glucose 6-phosphate and nicotinamide adenine dinucleotide phosphate (NADPH), accompanied in parallel by increased nucleotide synthesis. Furthermore, increased oxygen consumption rate (OCR)/extracellular acidification rate (ECAR), suggestive of metabolic dependence on glycolytic and PPP pathways, was detected in macroH2A1 KD cells [69]. Altogether, we demonstrated that macroH2A1 KD cells produced higher levels of acetyl-CoA; acetyl-CoA is employed as substrate in fatty acid synthesis, and this is reflected by the augmented amount of lipid droplets present in KD cells’ cytoplasm [68,69]. Integrated lipidomic and transcriptomic studies hinted to the fact that the central process leading to this change is most likely dependent on liver X receptors (LXRs) [69]. Interestingly the PPP and the LXRs pathway directly, and reciprocally, regulate the circadian clock [70,71,72]. Of note, whole body knock-out (KO) mice models for macroH2A1 display a NAFLD-like phenotype and derangements in glucose metabolism, dependent on the genetic background of the animals [73,74]. As NAFLD is a major risk factor for HCC insurgence, we hypothesized that large histone variants macroH2A1 might play a role in disease progression through regulating the expression of clock genes. In fact, overexpression, and consequent chromatin incorporation, of macroH2A1 proteins in hepatocytes or in HCC cells treated with free fatty acids lead to significant dysregulation in the expression of several clock-controlled genes involved in lipid metabolism (Figure 1) [74,75]. We have also shown that macroH2A1 KO mice present with abnormalities in circadian energy homeostasis [74]. Circadian clock and cell cycle are interdependent circadian oscillators, and how histone variants (H2A.Z, macroH2A1) can modulate these processes in a DNA-replication dependent fashion has just started to be unraveled [76,77]. A further layer of complexity is added by the fact that histone variants might compete for the same gene bodies occupancy [78], impacting on circadian clock and nutrient homeostasis in liver cells, predisposing them to tumorigenesis.

## 4. The Biological Clock and Systemic Therapy in Hepatocellular Carcinoma

The role of the biological clock in pharmaceutical intervention for the treatment of NAFLD and the effects of dietary habits changes, especially in relationship to time-of-day of food consumption, in the organism’s metabolic homeostasis and in the physiopathology of hepatic steatosis have been extensively and comprehensively described in a recent review [36], so here we will limit ourselves to mentioning some fundamental concepts regarding a potential role of meal timing and frequency scheduling in NAFLD-NASH-HCC progression. In the last two decades, numerous scientific studies have tried to clarify the role of the biological clock in the regulation of metabolic pathways, while little is known about the effects of metabolism intermediates on the functioning of the biological clock, especially in conditions of alterations of the metabolic homeostasis. For example, the restriction of nutritional intake to specific time windows, i.e., time restricted feeding (TRF), has direct effects on behavioral and physiological patterns and determines a response of the organism apt to anticipate the moment of possible food intake, a phenomenon called food anticipatory activity, whose regulatory centers and bio-molecular mechanisms are still poorly defined [79]. The effects of the temporal restriction of food intake are better known, especially in rodents when used as experimental models. Usually the fasting/feeding cycle corresponds temporally to the sleep/wake cycle, differing in the various animal species, which can be diurnal (active by day), nocturnal (active by night), or crepuscular (active especially during twilight, for example at dawn and sunset). When the availability of food is temporarily restricted and out of phase with the body’s natural cycles, the expression of circadian genes is also decoupled in the peripheral tissues with respect to the SCN, which will continue to be synchronized to the natural light/dark cycle. Furthermore, TRF was shown to have more favorable effects on physiological (weight, visceral fat), metabolic (glycemic levels, glucose tolerance), and hormonal (serum insulin and leptin levels) parameters with respect to ad libitum diet, even when the caloric intake was represented from a diet high in fat [80]. Another option is represented by intermittent/periodic fasting and fasting-mimicking diets, capable to bring on visceral fat decrease with no lean body mass change, hasten immune system renewal, hinder and partly undo bone mineral density loss, halt and reduce incidence of inflammatory diseases and cancer [81]. These data suggest the importance of the temporal characteristics of food intake as well as food abstinence or reduction in order to maintain an adequate energy balance, deriving from the equilibrium between energy intake and expenditure, which in turn corresponds to the sum of basal metabolism, energy expenditure for physical activity, and diet-induced thermogenesis [80,82,83,84,85,86,87]. As far as humans are concerned, the possibility of using time scheduled food intake, i.e., chrono-nutrition, as a therapeutic strategy in conditions of altered metabolism, such as obesity, metabolic syndrome, diabetes mellitus, liver steatosis, as well as NAFLD/NASH-related HCC is a recent proposal, even if knowledge on how timed feeding influences the circadian timing system and the molecular clockwork is still limited. Anyway, recent studies already suggest that the timing of nutrition could translate into a beneficial approach to improve weight loss and metabolic homeostasis in humans and support the possibility to combine timed feeding/fasting-associated interventions with standard therapeutic strategies for neoplastic diseases, liver cancer included, although the physiological mechanisms and molecular signaling pathways implicated in these favorable modifications need to be better defined [88].

## 5. Conclusions

Liver cancer, and in particular HCC, the most common histotype, is considered a foremost cause for cancer-related mortality worldwide. Apart from cases related to alcoholism and/or viral hepatitis, hepatocarcinogenesis actually establishes in the fibrosis/cirrhosis/inflammation background related to the NAFLD global epidemic. Hepatic steatosis and steato-hepatitis derive from deranged metabolic, autophagic, and inflammatory signaling pathways driven by the biological clock and interplay with time-related dynamics of gut microbiota and fasting-sleep/feeding-wake cycles. Chronodisruption, i.e., derangement of appropriate internal organization of physiological rhythmicity, is crucial in the genetic/epigenetic, metabolic, immunologic, and endocrine alterations involved in NAFLD/NASH/HCC pathogenic processes. Recent evidence suggests a potential role for chrononutrition over and above proper eating behavior in addition to well-established pharmacological interventions as future valuable therapeutic strategies.
